# Supplementary material for: A non-invasive method to directly quantify surface heterogeneity of porous materials
Source: Nat Commun. 2018 Feb 22;9:784. doi: 10.1038/s41467-018-03151-w (PMC5823877; doi:10.1038/s41467-018-03151-w)
Supplement: Supplementary file 1 — Supplementary Information [file 41467_2018_3151_MOESM1_ESM.pdf]

A non-invasive method to directly quantify surface heterogeneity of porous materials

Chiang *et al*.

Supplementary Note 1: Derivation of the generalized Porod's scattering law method (GPSLM)

Porod's law is commonly used to describe the scattering intensity, $I(Q)$, for two-phase systems with relatively smooth interfaces. At the large Q limit, it has been demonstrated by Porod that $I(Q)$ can be formulated as:

$$I(Q) \xrightarrow{Q\to\infty} 2\pi Q^{-4} \frac{1}{V} [(\Delta\rho)^2 S] \quad (1)$$

Q is the scattering wave vector, $\Delta\rho = \rho_1 - \rho_2$ is the contrast in scattering length density (SLD), where ρ_1 and ρ_2 are the SLD of phase 1 and phase 2, respectively. S and V are the total surface area and total volume seen by a neutron or X-ray beam.

For heterogeneous porous systems as described in Fig. 1 in the main text, more than two matrices are involved and these matrices have different SLDs. There are both inter-matrix and intra-matrix pores, which are empty under vacuum or are filled with probing fluid with SLD as ρ_f. There are interfaces between matrix 1 and pores, between matrix 2 and pores, and so on. Therefore, the SLD contrast depends on the particular matrix, and is the SLD difference between that matrix at one side and the probing fluid filled in pores at the other side of the interface. In other words, $\Delta\rho$ is a function of the interface, S, and $\Delta\rho(S) = \rho(S) - \rho_f$, where $\rho(S)$ is the SLD of the matrix at one side of the interface S. When there is no fluid in pores, $\rho_f = 0$.

For a system with heterogeneous surfaces, each interface between the matrix and the pore can be treated as a subsystem composed of two phases. The $[(\Delta\rho)^2 S]$ term in equation (1) changes with the surface S as $[(\Delta\rho(S))^2 S]$ and should be summed over all surface S for the heterogeneous materials. Therefore, the general equation for the Porod's scattering law should be written as an integral form as:

$$I(Q) \xrightarrow{Q\to\infty} 2\pi \langle\Delta\rho^2\rangle_s Q^{-4} \frac{S_T}{V} = C_{GPS} Q^{-4} \quad (2)$$

$\langle\Delta\rho^2\rangle_s$ is the mean square deviation of SLD, *MSD_{SLD}*, defined as

$$\langle\Delta\rho^2\rangle_s \equiv \frac{1}{S_T} \int (\rho(S) - \rho_f)^2 \, dS \quad (3)$$

S_T is the total interfacial area seen by neutrons or X-rays.

$C_{GPS} = 2\pi \langle\Delta\rho^2\rangle_s \frac{S_T}{V}$ is the generalized Porod's scattering constant and it is a function of ρ_f.

For neutron scattering, the SLD of probing fluid that fills the pores can be easily changed by either mixing the deuterated and hydrogenated solvents with different ratios or loading gases with different pressures. Changing the fluid SLD, ρ_f, simultaneously varies the MSD_{SLD}. It is reasonable to re-write $I(Q) = I(Q, \rho_f)$ for the contrast variation small-angle scattering experiment. Here, we use small-angle neutron scattering (SANS) experiments as examples. But the same method can be applied to small-angle X-ray scattering experiments too.

For the Q range when the scattering patterns follow the Porod's law as shown by equation (2), we define $IR(Q,\rho_f)$ as the ratio of SANS intensity at Q of the material with pore fluid SLD as ρ_f to that with pore fluid SLD as 0 (under vacuum). $IR(Q,\rho_f)$ is also equal to the ratio of the generalized Porod's scattering constant C_{GPS} between $\rho_f = \rho_f$ and $\rho_f = 0$, as following,

$$IR(Q,\rho_f) \equiv \frac{I(Q,\rho_f)}{I(Q,\rho_f=0)} \xrightarrow{Q\to\infty} \frac{2\pi\, Q^{-4}\frac{S_T}{V}\left[\frac{1}{S_T}\int\left(\rho(S)-\rho_f\right)^2\, dS\right]}{2\pi\, Q^{-4}\frac{S_T}{V}\left[\frac{1}{S_T}\int\left(\rho(S)-0\right)^2\, dS\right]} = \frac{C_{GPS}(\rho_f)}{C_{GPS}(\rho_f=0)} = \frac{\frac{1}{S_T}\int\left(\rho(S)-\rho_f\right)^2\, dS}{\frac{1}{S_T}\int\rho(S)^2\, dS} \quad (4)$$

In the Q range of Porod's scattering, equation (4) shows that $IR(Q,\rho_f)$ is independent of Q and we can express $IR(Q,\rho_f) = IR(\rho_f)$. For the data analysis of kerogens in the main text, we first fit the SANS data in the Q range that follows the generalized Porod's scattering using equation (2), obtain C_{GPS} at different pressures (corresponding to different fluid SLD, ρ_f), and then $IR(\rho_f)$ as a function of ρ_f can be calculated.

We define:

$$\rho_M{}^2 \equiv \frac{1}{S_T}\int\rho(S)^2\, dS \quad (5)$$

$\rho_M{}^2$ is integral over all interfaces and is the "surface average" of $\rho(S)^2$ in the heterogeneous material.

The last equality in equation (4) can be re-written as:

$$IR(\rho_f) = \frac{1}{S_T}\int\left(\frac{\rho(S)}{\rho_M}-\frac{\rho_f}{\rho_M}\right)^2\, dS = 1 - 2\frac{\rho_f}{\rho_M{}^2}\left(\frac{1}{S_T}\int\rho(S)\, dS\right) + \frac{\rho_f{}^2}{\rho_M{}^2} \quad (6)$$

Define

$$\rho_A \equiv \frac{1}{S_T}\int\rho(S)\, dS \quad (7)$$

ρ_A is also integral over all interfaces and is the "surface average" of SLD of the components at the interfaces, $\rho(S)$, in the heterogeneous material.

Equation (6) becomes

$$IR(\rho_f) = 1 - 2\frac{\rho_A}{\rho_M{}^2}\rho_f + \frac{\rho_f{}^2}{\rho_M{}^2} = 1 + \frac{\left(\rho_f-\rho_A\right)^2}{\rho_M{}^2} - \frac{\rho_A{}^2}{\rho_M{}^2} = \frac{\left(\rho_f-\rho_A\right)^2}{\rho_M{}^2} + \frac{\rho_M{}^2-\rho_A{}^2}{\rho_M{}^2} \quad (8)$$

Equation (8) shows that $IR(\rho_f)$ is a parabolic function of the pore fluid SLD, ρ_f. $IR(\rho_f)$ has the minimum of $\frac{\rho_M{}^2-\rho_A{}^2}{\rho_M{}^2}$ at $\rho_f = \rho_A$.

Define the normalized surface heterogeneity, Δ_H, as

$$\Delta_H{}^2 \equiv \frac{\rho_M{}^2-\rho_A{}^2}{\rho_M{}^2} = \frac{\left[\frac{1}{S_T}\int(\rho(S)-\rho_A)^2\, dS\right]}{\rho_M{}^2} \quad (9)$$

The last equality in equation (9) shows that ${\Delta_H}^2$ is an indicator of the degree that the SLDs of the components at all the interfaces deviate from the surface-averaged SLD, ρ_A. Moreover, Δ_H is an dimensionless parameter and can be compared for different materials.

From equation (8) and equation (9), we obtain:

$$IR_{min}(\rho_{f,min}) = {\Delta_H}^2 \tag{10}$$

$$\rho_{f,min} = \rho_A \tag{11}$$

$IR_{min}(\rho_{f,min})$ is the minimum of $IR(\rho_f)$ as a function of ρ_f. And $\rho_{f,min}$ is the pore fluid SLD when $IR(\rho_f)$ reaches the minimum.

Using equations (9)-(11), the surface properties of heterogeneous porous materials ρ_A, Δ_H, and ${\rho_M}^2$ can be obtained.

The *MSD_{SLD}*, $\langle \Delta\rho^2 \rangle_S$, can be expressed as:

$$\langle \Delta\rho^2 \rangle_S \equiv \frac{1}{S_T} \int \left(\rho(S) - \rho_f\right)^2 dS = {\rho_M}^2 - 2\,\rho_f\,\rho_A + {\rho_f}^2 \tag{12}$$

Using equations (2) and (12), the total interfacial area seen by neutrons, S_T, can be calculated by

$$S_T = \frac{I(Q)\,Q^4\,V}{2\pi\,\langle \Delta\rho^2 \rangle_S} = \frac{C_{GPS}\,V}{2\pi\,\langle \Delta\rho^2 \rangle_S} \tag{13}$$

$I(Q)$ and C_{GPS} in equation (13) should be obtained from the Q range that follows the generalized Porod's scattering law as described in equation (2).

It is also worth to mention that we choose $\rho_f = 0$ as the reference condition when calculating $IR(\rho_f)$ in equation (4). But this method can be extended to cases for any given value of ρ_f as the reference condition.

In the above derivations for GPSLM, the assumptions being used are listed as follows:

(1) There is a clear Porod's law scattering region (i.e. $I(Q) \sim Q^{-4}$) at relatively high Q region to include the scattering of all pore surfaces.
(2) All pores are accessible to the guest fluid.
(3) The SLD of the solid matrix does not change after loading guest fluid.

For the three assumptions listed above, only assumption (1) is related with the scattering feature from samples. Therefore, as long as the Porod's scattering law, i.e. equation (2), is valid, any material with structure described as assumptions (2) and (3) is able to apply GPSLM, even for the case where the pore structure and solid matrix structure are highly correlated. To demonstrate this point, a special case of the core-shell structure is used here as an example. In the core-shell system, the domains of heterogeneities, i.e. the core domain and the shell domain, are highly coupled and correlated. We demonstrate briefly here that even in this highly correlated system, the equation of Porod's scattering law shown in equation (2) is still valid, which means that GPSLM method works properly too from the scattering point of view.

The scattering of the core-shell model is well-known in the literature. Supplementary Figure 1 shows the simulated scattering intensity from a system composed of core-shell particles after convoluting with a realistic common instrument resolution function obtained from NG-7 SANS instrument in NIST Center for Neutron Research (NCNR). It should be noticed that all the measured experimental SANS intensity is the result of theoretical intensity convoluting with an instrument resolution function. All parameters used for this core-shell model are given in Supplementary Figure 1. It is clear that the exact total surface area S_T (known for the simulated system) and the experiment S_T (obtained from our GPSLM) are the same.

We can further prove theoretically that the Porod's scattering law shown in equation (2) works fine for the core-shell structure. For core-shell particles with inner core radius as R_c, outer radius as R_s, core SLD as ρ_c, and shell SLD as ρ_s, and immersed in fluid with SLD as ρ_f, the Fourier transformation of the particle can be written as $F_{core-shell}(Q, R_c, R_s, \rho_c, \rho_{in}, \rho_f) = F_c + F_s$, where $F_c = \left(\frac{4}{3}\pi {R_c}^3\right)(\rho_c - \rho_s)\frac{3j_1(QR_c)}{QR_c}$ and $F_s = \left(\frac{4}{3}\pi {R_s}^3\right)(\rho_s - \rho_f)\frac{3j_1(QR_s)}{QR_s}$ respectively.

The core-shell intra-particle structure factor can be written as $P_{core-shell}(Q, R_c, R_s, \rho_c, \rho_{in}, \rho_f) = |F_{core-shell}|^2 = {F_c}^2 + {F_s}^2 + F_c\, {F_s}^* + {F_c}^* F_s$. The scattering intensity of the core-shell system can be written as $I(Q) = n\, P_{core-shell}(Q, R_c, R_s, \rho_c, \rho_{in})$, where n is number density of the core-shell particles. For the simplicity reason, we can ignore the inter-particle structure factor here. It is straightforward to show that

${F_c}^2 \xrightarrow{Q\to\infty} 2\pi\, (\rho_c - \rho_s)^2 (4\pi\, {R_c}^2)\frac{1}{Q^4}$, which gives the surface area of inner core.

${F_s}^2 \xrightarrow{Q\to\infty} 2\pi\, (\rho_s - \rho_f)^2 (4\pi\, {R_s}^2)\frac{1}{Q^4}$, which gives the surface area of outer shell.

$F_c\, {F_s}^* + {F_c}^* F_s \xrightarrow{Q\to\infty} 0$ because the average intensity after convoluting with an instrument resolution function should be zero (a necessary step for all the measured scattering intensity).

This reduces to the general equation of the Porod's scattering law (equation (2)):

$$I(Q) \xrightarrow{Q\to\infty} 2\pi \left[\frac{1}{S_T}\int (\rho(S) - \rho_f)^2\, dS\right] Q^{-4}\frac{S_T}{V} = 2\pi\, \langle \Delta\rho^2 \rangle_s\, Q^{-4}\frac{S_T}{V} = C_{GPS}Q^{-4}$$

The above derivation is an exact theoretical result without any assumption. The only requirement is that the Q value should be sufficiently large so that the thickness of the core-shell particle $t \gg \frac{1}{Q}$.

Supplementary Note 2: SANS intensity calculation for model systems

The three model systems described in Supplementary Table 1 are designed to have very low total particle volume fraction, φ_T = 0.0015. The only reason to use the low volume fraction here is to simplify the calculation of the scattering intensity at low-Q as the structure factor $S(Q) \to 1$ for the full Q range. It should be noted that $S(Q) \to 1$ at high Q for any volume fraction, and therefore the existence of $S(Q)$ does not affect the Porod's scattering region no matter what

volume fraction is chosen here. Therefore, the conclusion demonstrated in the main text for theoretical model systems is true at any volume fraction.

The pores are the spaces between different particles. The SLDs for different particles are different. Hence, the SLD contrasts of the interfaces between particles and pores are different. The SANS intensity for these model systems can be expressed as:

$$I(Q,\rho_f) = \sum_i \left[n_i(\rho_i - \rho_f)^2 \left(\frac{4}{3}\pi R_i{}^3\right)^2 P_i(Q)\right] \quad (14)$$

$P_i(Q)$ is the form factor of spherical particle i.

$$P_i(Q) = \left[\frac{3}{QR_i}\left(\frac{\sin(QR_i)}{(QR_i)^2} - \frac{\cos(QR_i)}{QR_i}\right)\right]^2 \quad (15)$$

Supplementary Note 3: Structure stability of kerogens under pressure

The structure stability of the kerogen matrices under gas pressure is tested by investigating the structure change of kerogens after loading helium at the pressure of 31.1 MPa using SANS (Supplementary Figure 3a). Since helium has very small SLD even at pressure up to 31.1 MPa (SLD ≈ 2.3 $*10^{-7}$ Å^{-2})[1], it is transparent to neutrons and the change of scattering patterns before and after loading helium is due to the structural change of the kerogen itself. SANS curves without and with 31.1 MPa helium being loaded are almost identical for all of three kerogens (Supplementary Figure 3a). This indicates that solid matrices of the kerogens can maintain their structure under the pressure up to 31.1 MPa.

Supplementary Note 4: Notes for the calculations of surface properties of kerogens using SANS data

1) The density of methane inside the kerogen pores can be assumed to be the same as the bulk methane density at room temperature and pressure range being studied, i.e. $\rho_f = \rho_{CD_4}$.
   This is because that the Porod law region ($I(Q) \propto Q^{-4}$) in the SANS patterns of kerogens (Supplementary Figure 3) covers Q range at least from 0.012 Å^{-1} to about 0.03 Å^{-1} approximately corresponding to the pore size range from ≈ 200 Å to ≈ 500 Å. It has been shown that the confinement effect at this large length scale is negligible and the confined CD_4 gas density is expected to be close to the bulk gas density[2–4].
2) The SLDs of matrices in kerogens remain almost the same when filling kerogens with CD_4 gas.
   The kerogens have very small porosity based on N_2 isotherm measurements (Supplementary Table 3) and S_{GPS} is very close to S_{BET} (Supplementary Table 3 and Supplementary Note 5). These indicate that there are very few small pores (intra-matrix pores) with size approximately smaller than 200 Å in the kerogens (Supplementary Note 5). Therefore, the SLD change of pore walls due to gas penetration should be negligible.
3) All the pores with size approximately larger than 500 Å in the kerogens are accessible by methane gas based on the similar discussions in literature[5].

Supplementary Note 5: Calculation of specific surface area of kerogens with scattering patterns using the GPSLM

For the special case when kerogens are under vacuum, pore fluid SLD $\rho_f = 0$ and equation (12) reduces to

$$\langle \Delta\rho^2 \rangle_{s,vacuum} = {\rho_M}^2 \quad (16)$$

$\langle \Delta\rho^2 \rangle_{s,vacuum}$ is the MSD_{SLD} for sample under vacuum. ${\rho_M}^2$ can be calculated using equation (9):

$${\rho_M}^2 = \frac{{\rho_A}^2}{1-{\Delta_H}^2} \quad (17)$$

ρ_A and Δ_H can be directly obtained from the minimum of parabolic function $IR(\rho_f)$ as indicated by equations (10) and (11).

With known sample mass, m, and sample volume, V, inside the neutron beam and using equation (13), the specific surface area, i.e. the total interfacial area per mass of dry kerogen, calculated from the generalized Porod's scattering law method (GPSLM), S_{GPS}, can be expressed by:

$$S_{GPS} = \frac{S_T}{m} \quad (18)$$

Combining equations (13) and (16)-(18), S_{GPS} can be obtained by:

$$S_{GPS} = \frac{I(Q)_{vacuum}\, Q^4\, V}{2\pi\, {\rho_M}^2\, m} = \frac{C_{GPS,vacuum}\, V}{2\pi\, {\rho_M}^2\, m} \quad (19)$$

$I(Q)_{vacuum}$ and $C_{GPS,vacuum}$ are the absolute SANS intensity of kerogens measured under vacuum and C_{GPS} extracted at vacuum condition, respectively, and need to be obtained in the Q range that follows the generalized Porod's scattering law described in equation (2).

The extracted S_{GPS} is listed in Supplementary Table 3 together with the BET surface area measured by isotherm nitrogen adsorption, S_{BET}. For the most mature kerogen Sample 3, the highest CD_4 pressure that can be accessed by the current pressure device does not allow us to reach the minimum of $IR(\rho_f)$ used to extract ρ_A, Δ_H, and ${\rho_M}^2$ (see Fig. 4a in main text). We then fit $IR(\rho_f)$ curve of Sample 3 (Fig. 4a in main text) using equation (8) and determine $\rho_A \approx 3.42 * 10^{10}$ cm^{-2} and ${\rho_M}^2 \approx 1.2 * 10^{21}$ cm^{-4}. Using equation (19), $S_{GPS} \approx 11.12$ m^2/g is obtained for Sample 3.

The fact that SANS intensity data follow the generalized Porod's law in the region from about 0.012 Å^{-1} to 0.03 Å^{-1} indicates that only the surface of pores with their size approximately larger than $\frac{2\pi}{Q} \approx 20$ nm is counted into the total interfacial area seen by neutrons, S_T. N_2 with kinetic diameter of 380 pm is known to enter and detect pores much smaller than 20 nm. Therefore, $S_{BET} > S_{GPS}$ is expected as shown in Supplementary Table 3. The difference between S_{BET} and S_{GPS} gives the specific surface area contributed from pores with pore size approximately smaller 20 nm.

Supplementary Note 6: Accuracy of the average SLD and the surface area determined by traditional homogeneous analysis when heterogeneous surfaces are present in materials

Contrast variation has been widely used for extracting the "average SLD" of the scattering objects by assuming homogeneous two-phase system even though many systems have heterogeneous surface properties. Here, we apply the GPSLM method to quantitatively evaluate the effect of the surface heterogeneity on the values of ρ_A and S_T determined by the traditional Porod's scattering law method.

From equation (8):

$$IR(\rho_f) = \frac{(\rho_f - \rho_A)^2}{\rho_M{}^2} + \frac{\rho_M{}^2 - \rho_A{}^2}{\rho_M{}^2} = \frac{(\rho_f - \rho_A)^2}{\rho_M{}^2}\left[1 + \Delta_H{}^2 \frac{\rho_M{}^2}{(\rho_f - \rho_A)^2}\right] \quad (20)$$

The last equality in equation (20) indicates that the accuracy of determining ρ_A and surface area using the traditional homogeneous method depends on both Δ_H and the experimental data points of ρ_f used for the experiment. When $|\rho_f - \rho_A|$ is very large or Δ_H is very small, $IR(\rho_f)$ will reduce to the homogeneous case, i.e. $IR(\rho_f) \to \frac{(\rho_f - \rho_A)^2}{\rho_M{}^2}$.

Many papers have reported the average SLD values of heterogeneous systems. For example, Thomas *et al*[5] used hydrogenated methanol (CH_3OH with SLD ρ_{CH_3OH}= -0.374 * 10^{14} m^{-2}) and partially deuterated methanol (CD_3OH SLD ρ_{CD_3OH}= 4.284* 10^{14} m^{-2}) as fluids to determine the average SLD of many kerogen samples. By following similar method, we use kerogen Sample 1 with lowest maturity and highest surface heterogeneity to show the influence of surface heterogeneity on the value of average SLD and specific surface area obtained by both the traditional Porod's scattering law method and the GPSLM method.

We can calculate the generalized Porod's scattering constant, $C_{GPS}(\rho_f)$, using the GPSLM method at different ρ_f. $C_{GPS}(\rho_f)$ is proportional to the scattering contrast. The square root of intensity ratio, $IR(\rho_f)^{1/2}$, is calculated using $C_{GPS}(\rho_f)$, and plotted as a function of ρ_f for two contrast points determined by CH_3OH and CD_3OH in Supplementary Figure 4. (This is the common plot for contrast variation method for homogeneous system.) For the traditional Porod's scattering law method, $IR(\rho_f)^{1/2}$ is approximated as a linear function of ρ_f. The average SLD is normally found by the ρ_f at $IR(\rho_f)^{1/2}$= 0. $IR(\rho_f)^{1/2}$ for kerogen Sample 1 loaded with CH_3OH and CD_3OH (red circles in Supplementary Figure 4) is fitted linearly with ρ_f, i.e. ρ_{CH_3OH} and ρ_{CD_3OH} (see red linear line in Supplementary Figure 4). The average SLD found by assuming homogeneous system, $\langle\rho_{homo}\rangle$, is close to the real surface-averaged SLD, ρ_A. This is reasonable because both ρ_{CH_3OH} and ρ_{CD_3OH} are far away from ρ_A and the intensity at these two points can be approximated as homogeneous systems (equation (20)). Therefore, for this case, even though the traditional homogeneous analysis cannot give any information of the surface heterogeneity, it

is still fine to use the traditional method to obtain ρ_A even the system has heterogenous surface properties.

However, if the data with ρ_f close to ρ_A are used (blue triangles and blue linear fitting in Supplementary Figure 4), $\langle\rho_{homo}\rangle$ will be deviated much from ρ_A.

By assuming homogeneous system and using $\langle\rho_{homo}\rangle$ found in Supplementary Figure 4, the specific surface area, S_{homo}, can be estimated using equation (1), where $\Delta\rho = \langle\rho_{homo}\rangle$ for sample under vacuum. S_{homo} is 5.60 m^2/g and 4.64 m^2/g for homogeneous analysis using data with ρ_f close to ρ_A (red circles) and data with ρ_f far from ρ_A (blue triangles), respectively, compared with S_{GPS} = 3.85 m^2/g for GPSLM analysis without assumption of homogeneous system (Supplementary Note 5). Traditional Porod's method tends to overestimate the specific surface area in this case.

Supplementary Note 7: Hydrogen to carbon ratio (H/C) of the kerogen samples

The hydrogen atom density (hydrogen index) is related to R_0. We have done an experiment using the Prompt Gamma-ray Neutron Activation Analysis (PGAA) method to extract the hydrogen to carbon ratio (H/C) of the kerogen samples. The results are fully consistent with our results obtained by GPSLM method. H/C is decreased with R_0 as expected and agrees with the results reported by Thomas *et al*[5]. The Supplementary Table 5 lists the H/C ratio we determine from PGAA.

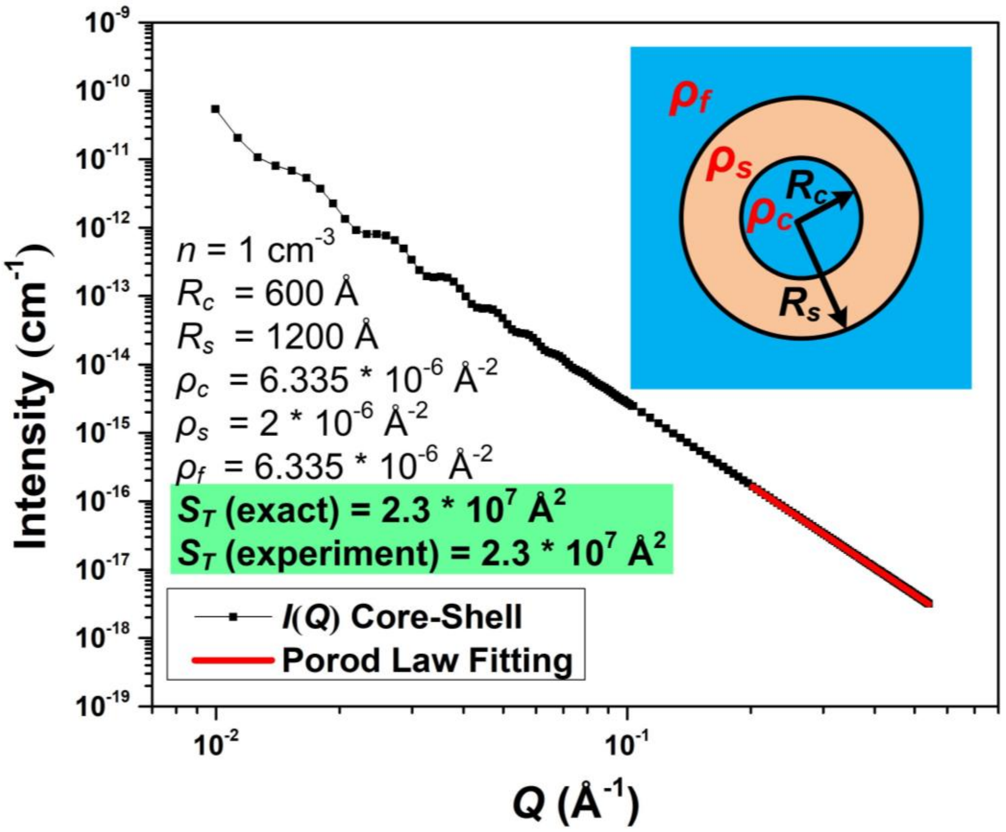

Supplementary Figure 1. The simulated scattering intensity for a system composed of core-shell particles after convoluting with instrument resolution function. The parameters for the core-shell particles are given in the figure. R_c, R_s, ρ_c, ρ_s, and ρ_s are inner core radius, outer radius, core scattering length density (SLD), shell SLD, and fluid SLD, respectively. The fitting to the Porod's law scattering region gives the total surface area S_T (experiment value) the same as the S_T calculated directly from the geometry of this particle (exact value).

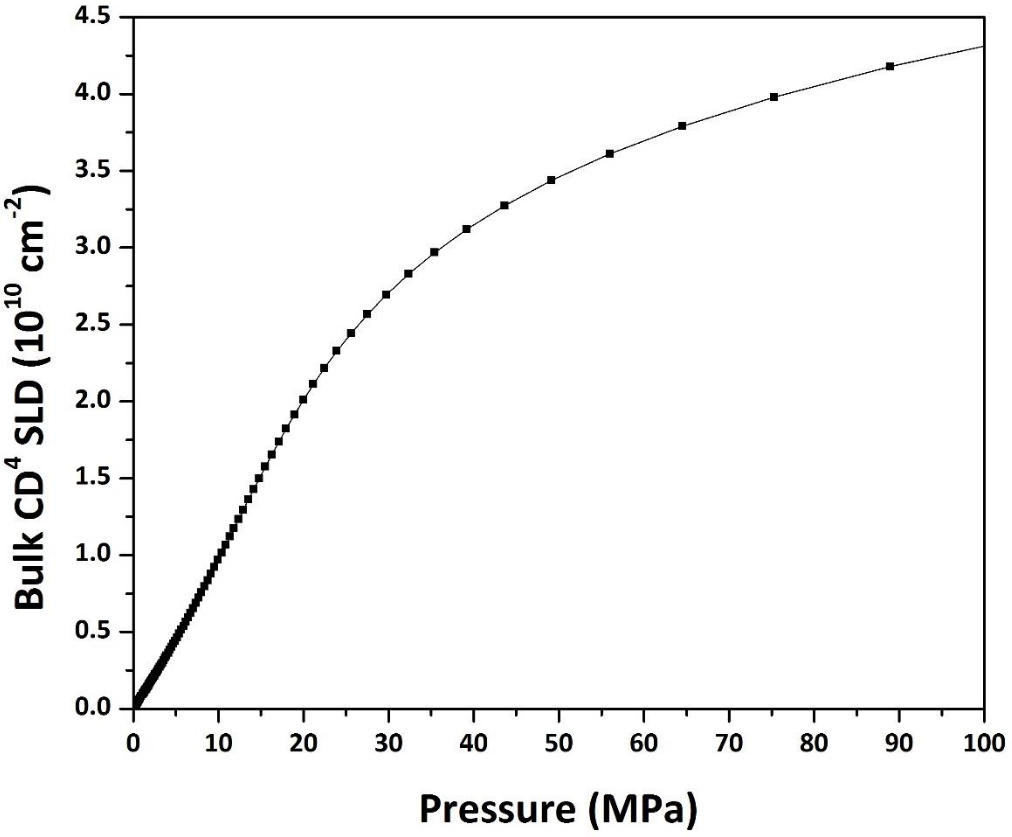

Supplementary Figure 2. Scattering length density (SLD) of bulk deuterated methane (CD_4) as a function of pressure. The SLD of bulk CD_4, ρ_{CD_4}, can be calculated by the formula $\rho_{CD_4} = \frac{\rho_{CD_4,mass}}{M_{w,CD_4}} N_A\, b_{CD_4}$, where $\rho_{CD_4,mass}$, M_{w,CD_4} = 20 g/mol, and b_{CD_4} = 3.33 * 10^{-4} Å are the mass density, molecular weight, and total coherent scattering length of CD_4 molecules. N_A is the Avogadro's constant. $\rho_{CD_4,mass}$ depends on pressure and is calculated using NIST standard reference database software REFPROP[1].

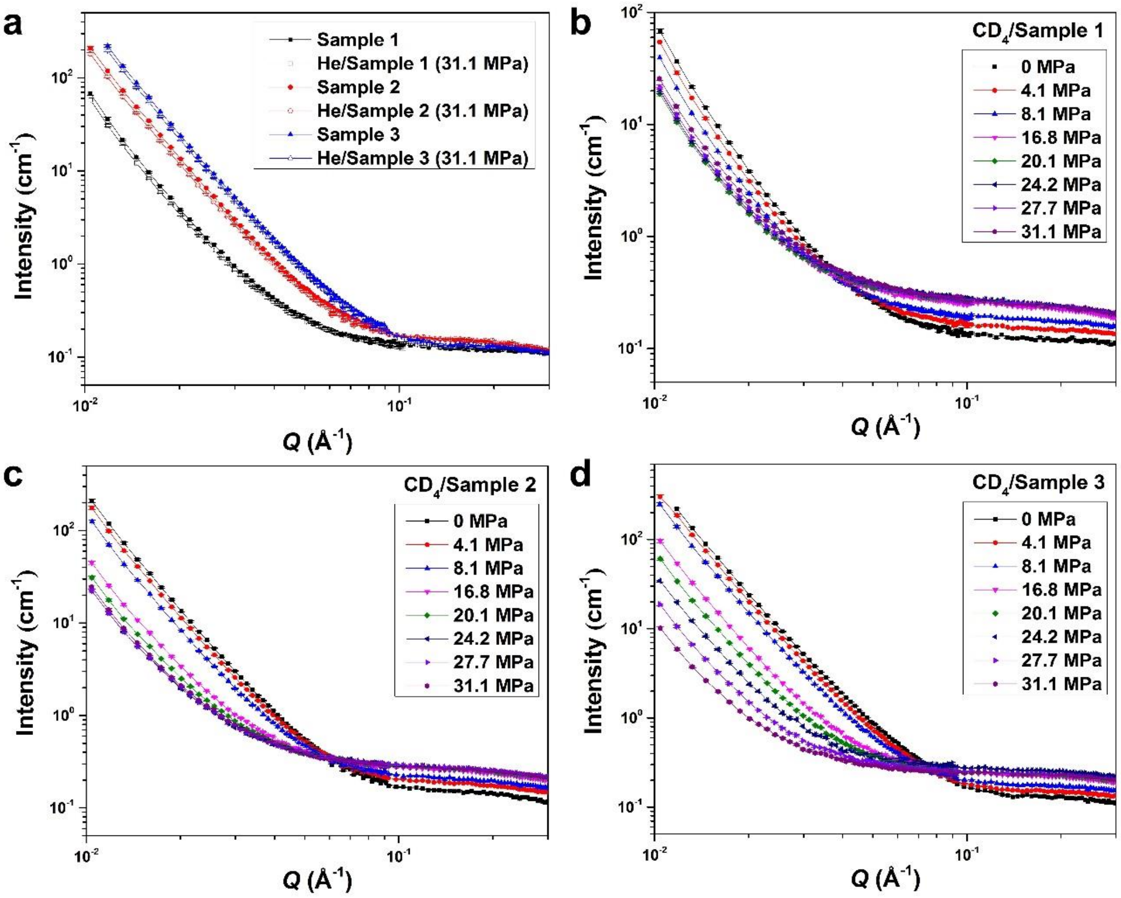

Supplementary Figure 3. Experimental data of small-angle neutron scattering (SANS) for gas loading kerogens. (a) Sample 1 (black squares, least mature), Sample 2 (red circles), and Sample 3 (blue triangles, most mature) at dry state (solid symbols) and loaded with 31.1 MPa helium (open symbols). (b) Sample 1 loaded with CD_4. (c) Sample 2 loaded with CD_4. (d) Sample 3 loaded with CD_4. CD_4 pressure is in the range of 0 to 31.1 MPa. At high *Q*, the intensity flattens off due to the incoherent scattering background contributed from both kerogens and CD_4. Error bars represent one standard deviation.

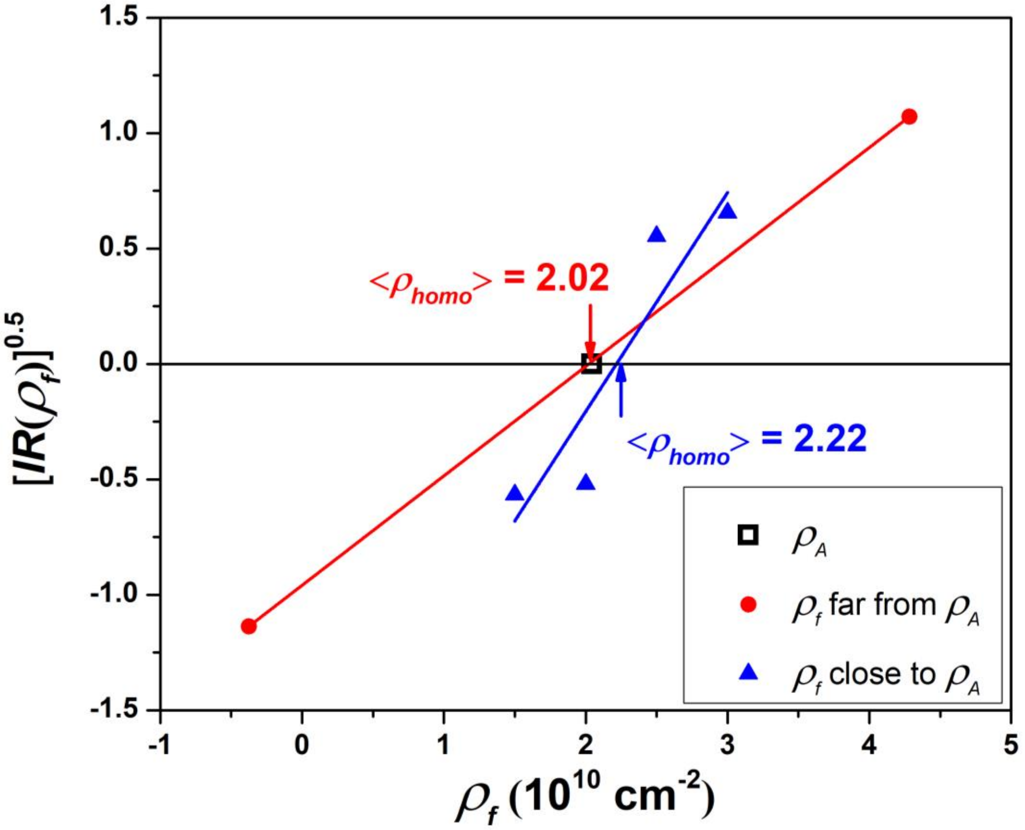

Supplementary Figure 4. The square root of intensity ratio, $IR(\rho_f)^{1/2}$, *versus* the guest fluid scattering length density (SLD), ρ_f, for kerogen Sample 1. The traditional contrast variation method of finding average SLD of the scattering object is to assume the system is a homogeneous system, where $IR(\rho_f)^{1/2}$ is a linear function of ρ_f, and assign the ρ_f at $IR(\rho_f)^{1/2} = 0$ to be the average SLD = $\langle\rho_{homo}\rangle$. Red line is the linear fitting result for data points (red solid circles) with ρ_f far away from real surface averaged SLD, ρ_A = 2.04 * 10^{10} cm^{-2} (black open square), that extracted from GPSLM. Blue line is the linear fitting result for data points (blue solid up-triangles) with ρ_f close to ρ_A. $\langle\rho_{homo}\rangle$ is the average SLD found from the linear fitting assuming homogeneous two-phase system.

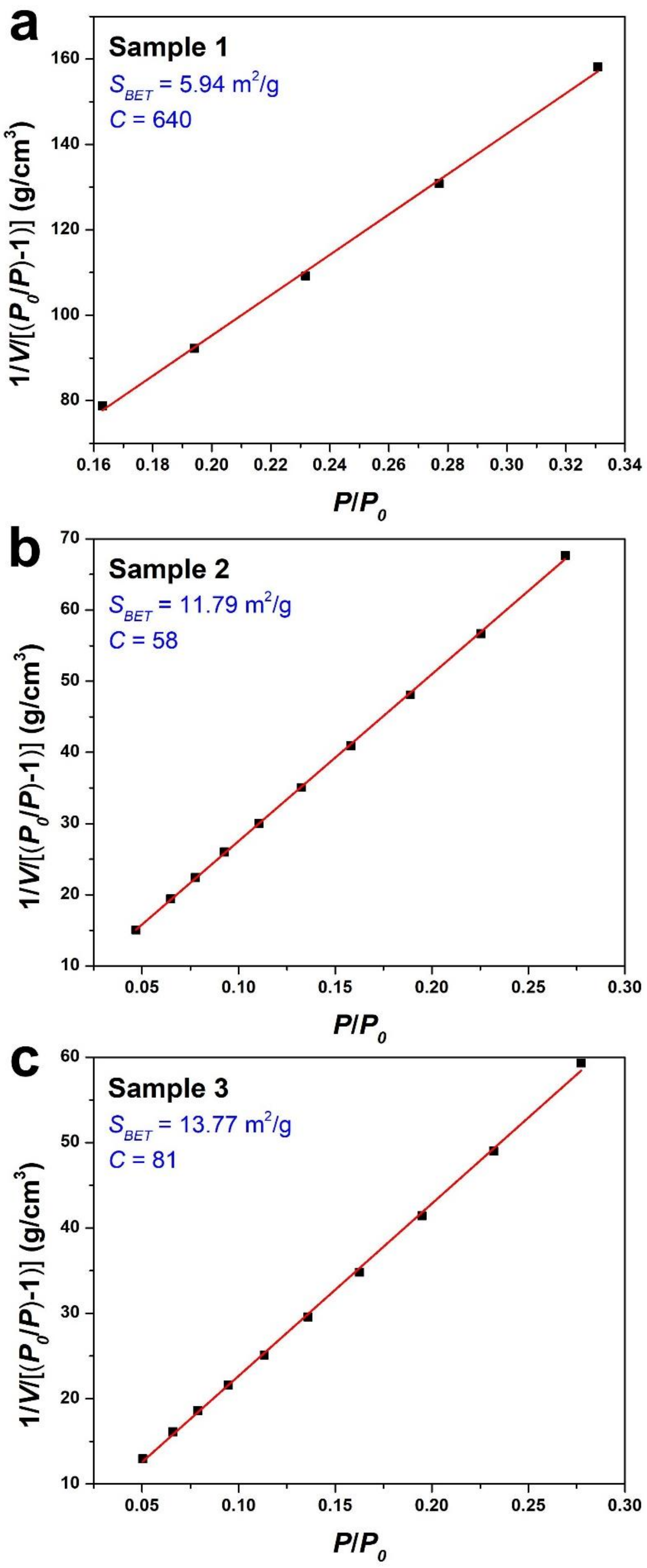
a
Sample 1
S_BET = 5.94 m2/g
C = 640
1/V[(P0/P)-1)] (g/cm3)
P/P0
b
Sample 2
S_BET = 11.79 m2/g
C = 58
1/V[(P0/P)-1)] (g/cm3)
P/P0
c
Sample 3
S_BET = 13.77 m2/g
C = 81
1/V[(P0/P)-1)] (g/cm3)
P/P0

Supplementary Figure 5. $\frac{1}{V[(\frac{P_0}{P})-1]}$ *vs.* $\frac{P}{P_0}$ for N_2 adsorption in kerogen (a) Sample 1 (least mature), (b) Sample 2, and (c) Sample 3 (most mature) at 77 K and the corresponding BET fitting (red line) using BET equation $\frac{1}{V[(\frac{P_0}{P})-1]} = \frac{C-1}{v_m C}\left(\frac{P}{P_0}\right) + \frac{1}{v_m C}$. P and P_0 are the equilibrium and the saturation pressure for N_2 at 77 K, V and v_m are the adsorbed volume and monolayer adsorbed volume for N_2, both in unit of cm^3 per gram of kerogen, and $C = \exp\left[\frac{E_1 - E_L}{RT}\right]$ is the BET constant.

Supplementary Table 1. Parameters of the particles composing the model systems used to examine the generalized Porod's scattering law method (GPSLM). These model systems are similar to the illustration shown in Figure 1 in the main text except that there are no intra-particle pores in these model systems in order to simplify the calculation of the scattering patterns. Inter-matrix pores are formed between the packed spherical particles.

Parameter	Model 1	Model 2	Model 3
R_1 (Å)	600	600	600
R_2 (Å)	1000	1000	1000
R_3 (Å)	800	800	800
R_4 (Å)	500	500	500
n_1 (Å^{-3})	4.00	3.00	0.90
n_2 (Å^{-3})	1.20	2.20	0.90
n_3 (Å^{-3})	1.80	0.30	3.50
n_4 (Å^{-3})	2.00	1.00	4.00
φ_T	0.0014	0.0013	0.0014
ρ_1 (10^{10} cm^{-2})	1.0	2.0	2.6
ρ_2 (10^{10} cm^{-2})	2.0	2.5	2.7
ρ_3 (10^{10} cm^{-2})	3.0	3.0	2.8
ρ_4 (10^{10} cm^{-2})	4.0	3.5	2.9
ρ_A (10^{10} cm^{-2})	2.166	2.448	2.788
ρ_M^2(10^{20} cm^{-4})	5.733	6.142	7.778
ΔH^2	0.182	0.024	0.001

R_i = radius of spherical particle *i*

n_i = number density of spherical particle *i* per 1 Å^3 volume

φ_T = total volume fraction of all the particles in the model

ρ_i = scattering length density (SLD) of spherical particle *i*

$$\rho_M{}^2 = \frac{1}{S_T}\int \rho(S)^2\, dS = \frac{\sum_i R_i^2\, \rho_i^2\, n_i}{\sum_i R_i^2\, n_i}$$

$\Delta_H{}^2 = \frac{\rho_M{}^2 - \rho_A{}^2}{\rho_M{}^2}$, where $\rho_A = \frac{1}{S_T}\int \rho(S)\, dS = \frac{\sum_i R_i^2\, \rho_i\, n_i}{\sum_i R_i^2\, n_i}$

Supplementary Table 2. True and extracted surface properties for model systems (see Supplementary Table 1).

System	ρ_A (10^{10} cm^{-2})	$\Delta_H{}^2$	$\rho_{f,min}$ (10^{10} cm^{-2})	$IR(\rho_{f,min})$
Model 1	2.166	0.182	2.17	0.182
Model 2	2.448	0.024	2.45	0.024
Model 3	2.788	0.001	2.79	0.001

$\rho_A = \frac{1}{S_T}\int \rho(S) dS$ = true surface averaged SLD (Supplementary Table 1).

$\rho_f = \frac{\rho_M{}^2 - \rho_A{}^2}{\rho_M{}^2}$ = true normalized surface heterogeneity (Supplementary Table 1).

$\rho_{f,min}$= the fluid SLD where the minimum of $IR(\rho_f) = \frac{I(Q,\rho_f)}{I(Q,\rho_f = 0)}$ takes place (see Fig. 2b in main text).

$IR(\rho_{f,min})$ = the minimum value of $IR(\rho_f)$ (see Fig. 2b in main text).

Supplementary Table 3. Properties of kerogens. BET surface area and the specific surface area extracted from GPSLM (Supplementary Note 1 and Supplementary Note 5), S_{GPS}, S_{BET}, and pore volume are obtained from N_2 volumetric adsorption isotherm measurement at 77 K. Pore volume is calculated from the total amount of N_2 condensed in the samples at P/P_0 = 0.98 together with the bulk mass density of liquid N_2 at 77 K.

Sample	Vitrinite Reflectance (%)	S_{BET} (m^2/g)	Pore Volume (cm^3/g)	BET *C* constant	S_{GPS} (m^2/g)
Sample 1	0.62	5.94	0.06	640	3.85
Sample 2	1.02	11.79	0.07	58	8.44
Sample 3	1.59	13.77	0.07	81	11.12

BET *C* constant = exp $\left[\frac{E_1 - E_L}{RT}\right]$, where E_1 the heat of adsorption for the first layer and E_L is the heat of adsorption for the second and higher layers. High *C* value for the kerogens, especially for Sample 1, suggesting large amount of micropores in the kerogens.

Supplementary Table 4. Parameters of kerogens extracted from GPSLM. The SANS data with Q range that follows equation (2) are used for GPSLM analysis (≈ 0.01 $Å^{-1} < Q <$ 0.03 $Å^{-1}$).

Sample	ρ_A (10^{10} cm^{-2})	${\rho_M}^2$ (10^{20} cm^{-4})	$\sqrt{{\rho_M}^2}$ (10^{10} cm^{-2})	Δ_H	S_{GPS} (m^2/g)
Sample 1	2.043	5.71 ± 0.05	2.39 ± 0.01	0.519 ± 0.006	3.85
Sample 2	2.346	6.06 ± 0.02	2.462 ± 0.004	0.302 ± 0.004	8.44
Sample 3	2.764	7.831 ± 0.005	2.7984 ± 0.0009	0.157 ± 0.002	11.12

ρ_A is CD_4 SLD (= ρ_f) that gives the minimum $IR(\rho_f)$.

${\Delta_H}^2$ is found by the value of minimum of $IR(\rho_f)$, $IR(\rho_{f,min})$.

${\rho_M}^2 = \frac{{\rho_A}^2}{1-{\Delta_H}^2}$.

S_{GPS} is found by the procedure described in Supplementary Note 5.
For Sample 3, the highest pressure available for current experimental setup is not high enough to reach minimum $IR(\rho_f)$, the ρ_A, ${\rho_M}^2$, $\sqrt{{\rho_M}^2}$, ΔH, and S_{GPS} are approximate values based on the information from the highest pressure measurement.

Supplementary Table 5. Hydrogen carbon ratio (H/C) determined by the PGAA experiment of kerogen samples studied in the main text.

Sample ID	R_0 (%)	H/C
Sample 1	0.62	1.02(4)
Sample 2	1.02	0.93(4)
Sample 3	1.59	0.63(3)

Supplementary References

1. Lemmon, E.; Huber, M.; McLinden, M. No Title. *REFPROP, NIST Stand. Ref. Database 23, Version 9.0*

2. Eberle, A. P. R. *et al.* Direct Measure of the Dense Methane Phase in Gas Shale Organic Porosity by Neutron Scattering. *Energy & Fuels* **30,** 9022–9027 (2016).

3. Chiang, W.-S., Fratini, E., Baglioni, P., Chen, J.-H. & Liu, Y. Pore Size Effect on Methane Adsorption in Mesoporous Silica Materials Studied by Small-Angle Neutron Scattering. *Langmuir* **32,** 8849–8857 (2016).

4. Chiang, W.-S. *et al.* Methane Adsorption in Model Mesoporous Material, SBA-15, Studied by Small-Angle Neutron Scattering. *J. Phys. Chem. C* **120,** 4354–4363 (2016).

5. Thomas, J. J., Valenza, J. J., Craddock, P. R., Bake, K. D. & Pomerantz, A. E. The neutron scattering length density of kerogen and coal as determined by CH3OH/CD3OH exchange. *Fuel* **117,** 801–808 (2014).
